# Expressions of CD8+TILs, PD-L1 and Foxp3+TILs in stage I NSCLC guiding adjuvant chemotherapy decisions

**DOI:** 10.18632/oncotarget.11793

**Published:** 2016-09-01

**Authors:** Feifei Teng, Xiangjiao Meng, Xin Wang, Jupeng Yuan, Sujing Liu, Dianbin Mu, Hui Zhu, Li Kong, Jinming Yu

**Affiliations:** ^1^ Department of Radiation Oncology, Shandong Cancer Hospital and Institute, Shandong University, Jinan, China; ^2^ Department of Pathology, Shandong Cancer Hospital and Institute, Shandong University, Jinan, China; ^3^ Shandong Provincial Key Laboratory of Radiation Oncology, Shandong Cancer Hospital and Institute, Shandong University, Jinan, China

**Keywords:** non-small cell lung cancer, TILs, PD-L1, Foxp3, adjuvant chemotherapy

## Abstract

**Purpose:**

Currently, adjuvant chemotherapy is recommended for patients with high risk stage I non-small cell lung cancer (NSCLC). However, identifying high risk patients remains a challenge. This study aims to identify the patient cohorts more likely to benefit from adjuvant chemotherapy based on the tumor micro-immune environment.

**Results:**

CD8+TILs significantly associated with disease-free survival (DFS) and overall survial (OS) (p=0.002; 0.040). Patients with high risk factors may also predict shorter DFS (P=0.056). When compared together, patients with high-CD8+TILs showed better DFS than patients with low-CD8+TILs, no matter their risk factors status. There's no correlation between PD-L1 expressions and survival. PD-L1 was highly expressed in men, squamous and well differentiated carcinoma. In addition, Foxp3+TILs alone didn't show any prognostic effects, but low-Foxp3/high-CD8+TILs were associated with prolonged DFS (p=0.031).

**Methods:**

A total of 126 patients with surgically resected stage I NSCLC were included to perform immunohistochemistry of CD8+ tumor infiltrating lymphocytes (TILs), programmed death ligand-1(PD-L1) and forkhead box P3 (Foxp3)+TILs.

**Conclusion:**

CD8+TILs are effective prognostic predictors. Patients with surgically resected stage I NSCLC showing low CD8+TILs could be considered for adjuvant chemotherapy, even if they have no high risk features.

## INTRODUCTION

Lung cancer remains the leading cause of cancer deaths worldwide [1]. Non-small-cell lung cancer (NSCLC)accounts for 80%–85% of all cases [2]. Complete surgical resection is the primary therapeutic approach for early-stage NSCLC. Recently, some studies reported that stereotactic body radiotherapy could be an alternative option for inoperable or operable stage I NSCLC [3]. However, at least around 30% patients still experienced recurrence within 5 years, suggesting that many stage I NSCLC might be subclinical metastatic at the time of diagnosis [4]. To improve patient outcomes, cisplatin-based adjuvant chemotherapy is recommended for early-stage patients with high-risk features, such as poor histologic differentiation, vascular invasion, wedge resection, tumors >4 cm, incomplete lymph node sampling and visceral pleural invasion [5]. Agassi et al. [6]reported that these factors may not be independently significant indications but might be considered when determining adjuvant chemotherapy. Even with adjuvant chemotherapy stage I patients still have a significant locoregional or systemic recurrence and lung cancer death [7]. The reported 5-year survival rates of patients with stage I tumors are approximately 60-90% [8]. The effects of chemotherapy on survival are limited and may be unsuitable for some patients due to toxicity. Considering the benefits and toxicity, debates still exist surrounding which population of patients is most likely to achieve optimal outcomes from chemotherapy [9]. Currently, adjuvant chemotherapy is recommended for patients with high risk stage I non-small cell lung cancer (NSCLC) [5]. However, the NCCN didn't clarify the details. Previous literatures reported that patients with more than three risk factors should be given adjuvant chemotherapy. In contrast, patients with stage I disease with either zero or one risk factor had a favorable outcome. Adjuvant chemotherapy may not be necessary for these patients [5]. Till now, how to identify these high risk patients remains challenging.

Tumor-infiltrating lymphocytes (TILs), especially CD8+ cytotoxic T-lymphocytes (CD8+TILs), have been associated with better survival in many forms of cancers, including NSCLC [10, 11]. However, because lymphocytes are very sensitive to chemotherapy and radiotherapy, the predictive effects of TILs in NSCLC remains controversial, which may be due to the different therapeutic regimens patients received across studies. What's more, T-cell activation is controlled by a group of immune checkpoint signals. Program death ligand 1 (PD-L1), which is often over-expressed in many tumors, is involved in negative regulation of immune responses through exhaustion of CD8+T cells [12]. Previous studies find that PD-L1 expressions are associated with tumor grade and prognosis in colorectal, renal, bladder and lung cancers [13–16]. However, the predictive effects of PD-L1 in NSCLC remain controversial and unclear, especially in early stage patients [17–20]. Besides immune checkpoints, some studies find that the forkhead box P3 (Foxp3)+ regulatory T-cell (Treg) is the only statistically significant lymphocyte which indicated higher recurrence rates for NSCLC [21, 22]. Treg is characterized by expression of Foxp3 and plays an important role in the cancer immunosuppressive mechanism [23]. Foxp3+T cells account for 5–10% of the total CD4+ T cells and inhibit human anticancer activities through diminishing the activation of lymphocytes [24].

Tumor molecular features have become more prevalent in recent years for diagnosis and treatment planning. Pathological immunity evaluation can provide new and meaningful information on prognosis. Previous studies have demonstrated that adaptive immune cell and immune markers within tumor regions are strongly associated with response to therapy and prognosis, which may add to the significance of the TNM-classification [25]. Even more, these immune parameters may be more competitive as biomarkers to classify cancers and guide the following treatment regimens than traditional classifications [25]. In our studies, we aim to discuss CD8+TILs, PD-L1 and Foxp3+T cells expressions in resected stage I NSCLC and their associations with recurrence and prognosis. We are going to identify the patient population more likely to benefit from adjuvant chemotherapy based on the tumor micro-immune environment.

## RESULTS

### Patient characteristics

We analyzed CD8+TILs, PD-L1 and Foxp3+TILs expressions in 126 patients with stage I NSCLC. To eliminate the effects of chemotherapy or radiotherapy to recurrence outcomes, all patients enrolled only received surgical resection without any neoadjuvant or adjuvant chemotherapy and radiotherapy. Patient characteristics are summarized in Table 1. The median age is 61 years (range 38–78 years) and 67% (n = 85) are men. There are 33% patients with squamous carcinoma, 45% patients with adenocarcinoma and 22% patients with large cell lung cancers. Tumor differentiation grade is classified as either poor (24%), moderate (47%) or well differentiated (29%). There are 56% patients with high risk factors, including histologic differentiation, vascular invasion, wedge resection, tumors >4cm, incomplete lymph node sampling or visceral pleural invasion [6].

**Table 1:** Clinicopathological characteristics of 126 patients

Clinicopathological parameters	Cases (%)
Age	
≤ 60	61(48)
> 60	65(52)
Sex	
Male	85(67)
Female	41(33)
Histology	
Squamous	42(33)
Adenocarcinoma	57(45)
Large Cell	27(22)
Differentiation	
Poor	30(24)
Moderate	59(47)
Well	37(29)
High Risk	
Yes	70(56)
No	56(44)

### CD8+TILs, PD-L1 and Foxp3+TILs expressions and correlations with clinical characteristics

**Figure 1:**
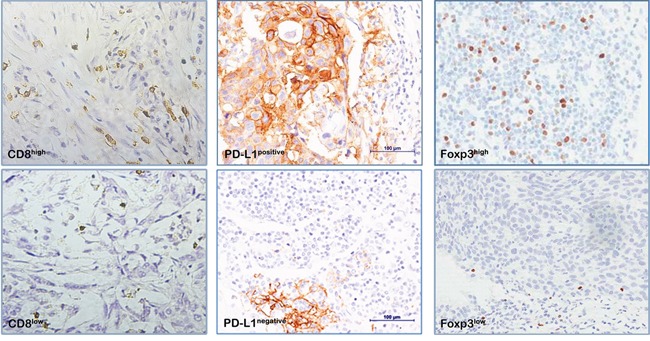
Immunohistochemical staining for immune markers in NSCLC cells (400x) CD8, cluster of differentiation 8; Foxp3, transcription factor forkhead; PD-L1, programmed death ligand-1.

As shown in Figure 1, CD8 are clearly stained in the cell membrane of interstitial infiltrates. The median value of CD8+TILs is 30%, there are 71 patients defined as high CD8+TILs (≥30%) and 55 patients defined as low CD8+TILs (<30%). The expression of CD8+TILs has no significant relationships with age, gender, histology, high risk factor and tumor differentiation. (Table 2)

**Table 2:** Correlations between immune markers expressions and clinicopathologic characteristics

		High CD8+TILs	P value	High PD-L1	P value	High FOXP3+TILs	P value
Age	≤ 60	33/61(54.0%)	0.622	11/61(18.0%)	0.622	32/61(52.4%)	0.161
	> 60	38/65(58.4%)		14/65(21.5%)		26/65(46.4%)	
Sex	Male	49/85(57.6%)	0.672	24/85(28.2%)	<0.001*	42/85(72.4%)	0.273
	Female	22/41(53.6%)		1/41(2.4%)		16/41(39.0%)	
Histology	Squamous	23/42(54.7%)	0.736	17/42(40.4%)	<0.001*	23/42(54.7%)	0.018*
	Adenocarcinoma	31/57(54.4%)		4/57(7.0%)		29/57(50.8%)	
	Large Cell	17/27(66.7%)		4/27(14.8%)		6/27(22.2%)	
Differentiation	Poor	18/30(60%)	0.873	10/30(33.3%)	0.013*	15/30(50%)	0.737
	Moderate	32/59(54.2%)		13/59(22.0%)		25/59(42.3%)	
	Well	21/37(56.7%)		2/37(5.4%)		18/37(48.6%)	
High Risk	Yes	38/70(54.2%)	0.807	12/70(17.1%)	0.369	29/70(41.4%)	0.246
	No	33/56(58.9%)		13/56(23.2%)		29/56(51.7%)	
CD8+TILs	High	—		17/71(23.9%)	0.244	35/71(49.2%)	0.578
	Low	—		8/52(15.3%)		23/52(44.2%)	
PD-L1	High			—		16/25(64%)	0.059
	Low			—		42/98(42.8%)	

* Statistically significant

Distinct positive staining of PD-L1 was found in cell membrane of both tumor cells and stroma cells. In 126 early stage NSCLC patients, there are 19.8% (n=25) patients showing positive PD-L1 expression (≥5%). PD-L1 expression is higher in men than women (28.2% vs. 2.4%, p<0.001). Patients with squamous carcinoma show significantly higher PD-L1 expression than adenocarcinoma and large cell lung cancers (40.4% vs. 7.0% vs. 14.8%, p<0.001). Moreover, PD-L1 expression has strong associations with tumor differentiation. Poor differentiated tumor tends to have higher PD-L1 expressions than moderate and well differentiated tumor (33.3% vs. 20.0% vs. 5.4%, p=0.013). There are no significant correlations between PD-L1 expression and age, high risk factors or CD8+TILs density.

Foxp3 positive staining is located in cell nuclei. Different densities of Foxp3+TILs were found in the stroma surrounding the tumor cells. The median count of Foxp3+TILs is 45/HPF. Patients were divided into Foxp3-high group (≥45/HPF) and Foxp3-low group (<45/HPF). There are no differences in Foxp3 expression by age, gender, tumor differentiation, high risk factors, CD8+TIL density or PD-L1 expressions, except histology. Squamous carcinoma and adenocarcinoma show increased Foxp3 expressions than large cell tumors (54.7% vs. 50.8% vs. 22.2%, p=0.018).

### Survival analysis

**Figure 2:**
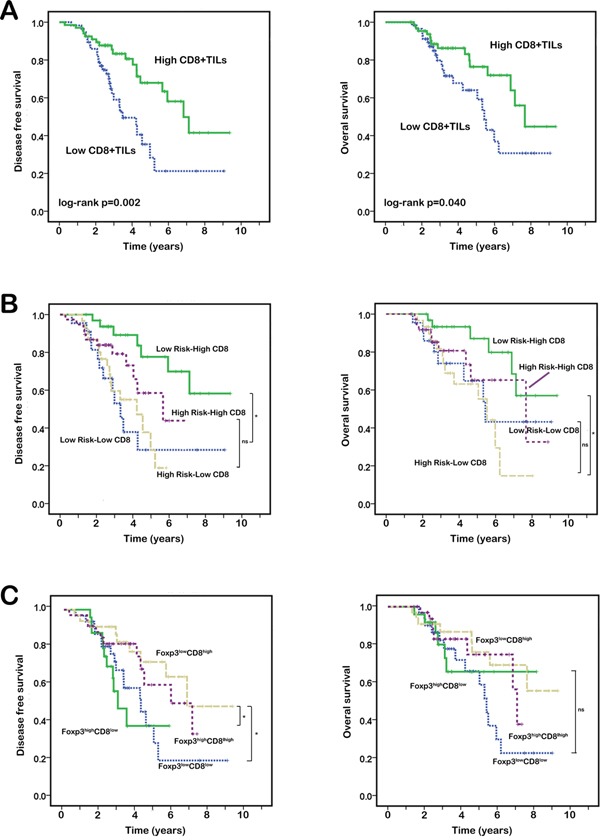
Kaplan–Meier analysis of DFS and OS according to CD8+TILs, high risk features and Foxp3+TILs in NSCLC **A.** Patients with high CD8+TILs achieved longer DFS and OS than patients with low CD8+TILs. **B.** Patients with low-risk/high-CD8+TILs had significant better DFS than other 3 groups (P<0.05). Although there were no significant differences in other 3 groups, patients with high-risk/high-CD8+TILs seemed show better DFS than patients in other two low-CD8+TILs groups even they show low risk features. In the analysis of OS, patients with low-risk/high-CD8+TILs showed significant longer OS than patients with high-risk/low-CD8+TILs (p=0.003). The differences between other groups were not statistically significant. **C.** Patients with low-Foxp3/high-CD8+TILs showed better DFS than patients with low-Foxp3/low-CD8+TILs and high-Foxp3/low-CD8+TILs (p=0.048 and 0.017, respectively). No significant differences between these 4 groups were found in the analysis of OS.

The median follow up duration of the study is 80 months. To determine the prognostic effect of these clinicopathologic characteristics and immune markers, univariate and multivariate analysis were used. As shown in Table 3, high CD8+TILs are significantly associated with better DFS (HR, 0.393; 95% CI, 0.217-0.714; P=0.002). It seems that patients with high risk factors might have shorter DFS, though the association is not statistically significant (HR, 1.794; 95% CI, 0.986-3.263; P=0.056). In addition, though Foxp3+TILs alone haven't show any prognostic effects, the combination of Foxp3+TILs and CD8+TILs are significant predictors of DFS (HR, 0.760; 95% CI, 0.592-0.976; P=0.031). Patients with low-Foxp3/high-CD8+TILs (low-Foxp3 and high-CD8+TILs) tend to have prolonged DFS. (Figure 2C) However, we think that the prognostic value of Foxp3/CD8+TILs might mostly due to the effect of CD8+TILs. In the DFS analysis, there was no difference between patients with low-Foxp3/low-CD8+TILs and high-Foxp3/low-CD8+TILs (p=0.555). In addition, no difference was found between patients with low-Foxp3/high-CD8+TILs and high-Foxp3/high-CD8+TILs (p=0.501). Age, gender, histology, tumor differentiation, PD-L1 expressions, Foxp3+TILs and peripheral circulated lymphocytes are not associated with DFS (p>0.05). In multivariate analysis, CD8+TILs density is an independently significant predictor for longer DFS (HR, 0.218; 95% CI, 0.053-0.892; P=0.034). Using univariate analysis, only CD8+TILs expression is associated with OS (HR, 0.505; 95% CI, 0.259-0.982; P=0.044). High risk features also show the trend toward predictive effects for OS. However, the p value is not statistically significant (HR, 1.947; 95% CI, 0.992-3.820; P=0.053). The prognostic nomograms that integrated possible factors for DFS and OS are shown in Supplementary Figure S2.

**Table 3:** Factors associated with DFS and OS in univariate and multivariate analyses

	DFS						OS					
	Univariate analysis			Multivariate analysis			Univariate analysis			Multivariate analysis		
	HR	95%CI	P value	HR	95%CI	P value	HR	95%CI	P value	HR	95%CI	P value
Age	0.985	0.953- 1.018	0.363				0.980	0.944- 1.017	0.278			
Gender	1.400	0.749- 2.618	0.291				1.427	0.697- 2.921	0.331			
Histology	1.212	0.845- 1.740	0.296				1.306	0.877- 1.945	0.189			
Differentiation	1.227	0.819- 1.838	0.322				1.153	0.716- 1.858	0.559			
High risk	1.794	0.986- 3.263	0.056	1.565	0.835- 2.934	0.162	1.947	0.992- 3.820	0.053	1.885	0.935- 3.481	0.145
CD8+TILs	0.393	0.217- 0.714	0.002*	0.218	0.053- 0.892	0.034*	0.505	0.259- 0.982	0.044*	0.539	0.276- 1.052	0.070
PD-L1	0.975	0.494- 1.925	0.943				0.999	0.467- 2.136	0.998			
FOXP3+TILs	1.302	0.727- 2.331	0.375				0.821	0.413- 1.632	0.574			
FOXP3/CD8+TILs^#^	0.760	0.592- 0.976	0.031*	1.370	0.758- 2.476	0.297	0.780	0.584- 1.043	0.094			
Circulating Lymphocytes	1.214	0.685- 2.125	0.506				1.683	0.863- 3.283	0.126			

* Statistically significant

# low-Foxp3 and high-CD8+TILs

As shown in Figure 2A, patients with high CD8+TILs show better DFS and OS (log-rank p=0.002; 0.040). To identify the population most likely to gain benefits from adjuvant chemotherapy, we compared the prognostic effects of CD8+TILs and risk factor status. The patients were divided into the following 4 groups: (1) patients with high risk and high CD8+TILs; (2) patients with high risk and low CD8+TILs; (3) patients with low risk and high CD8+TILs; and (4) patients with low risk and low CD8+TILs. As shown in Figure 2B, patients in the low-risk/high-CD8+TILs group show the best DFS.(p<0.05) Patients with high-risk/high-CD8+TILs show better DFS than patients with low-risk/low-CD8+TILs and high-risk/low-CD8+TILs, although the difference is not statistically significant. Patients with high-CD8+TILs show better DFS than patients with low-CD8+TILs, no matter their risk factor status. In the analysis of OS, the overall survival of patients with low-risk/high-CD8+TILs is longer than others. (p<0.05).

### The associations between CD8+TILs and peripheral circulating lymphocytes

**Figure 3:**
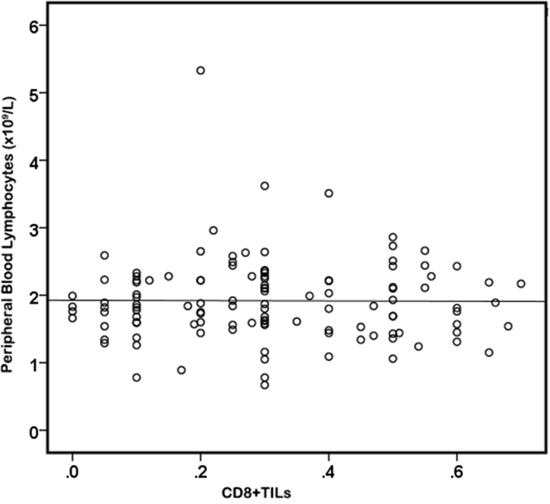
There's no significant correlation between CD8+TILs and peripheral blood lymphocytes (r=−0.066; p=0.466).

To identify the associations between CD8+TILs and circulated lymphocytes, Pearson's correlation analysis was used. As shown in Figure 3, there is no significant correlation between peripheral blood lymphocytes and CD8+TILs (r=−0.066; p=0.466).

## DISCUSSION

There are still some debates surrounding the use of adjuvant chemotherapy in patients with stage I NSCLC. In this study, we found that CD8+TILs were significantly associated with DFS and OS in patients with surgically resected stage I NSCLC. What's more, patients with high-CD8+TILs show better DFS than patients with low-CD8+TILs, whatever the risk status. This may indicate that patients with low CD8+TILs should be considered for adjuvant chemotherapy even if they don't have traditional high risk features. Anti PD-1/PD-L1 antibody has been applied in NSCLC widely. As a potential predictive marker, PD-L1 expressions in stage I NSCLC remain controversial. In our present study, we demonstrated that there is no correlation between PD-L1 expressions and patient outcomes. PD-L1 tended to be highly expressed in men, squamous and well differentiated carcinoma. In addition, as a group of immunosuppressive lymphocytes, Foxp3+TILs alone did not show any prognostic effects in patients with stage I NSCLC, but patients with low-Foxp3/high-CD8+TILs tended to have prolonged DFS.

Previous clinical trials and meta-analysis have demonstrated that adjuvant chemotherapy did not show survival benefits on average in patients with stage I NSCLC [26, 27]. However, the recurrence rate in stage I patients remains relatively high, which may indicate that there is a subpopulation of patients that would benefit from additional treatment. Currently, adjuvant chemotherapy is recommended for patients with early stage tumors that have high risk factors. However, identifying these high risk patients remains a challenge [5]. Considering the toxicity and cost of chemotherapy, it is crucial to identify patients who are most likely to achieve optimal outcomes from adjuvant chemotherapy. In our study, CD8+TILs showed better predictive effects on prognosis than high risk features. It indicates that CD8+TILs expression is an effective biomarker to guide adjuvant chemotherapy, which may add to the significance of the traditional high risk features. Since 2006, Galon et al [10]. have demonstrated that TILs may be a potential prognostic tool in the treatment of colorectal cancer. The correlation between TILs and patient survival has been well reported in melanoma, head and neck, ovarian, breast, urothelial, colorectal, lung, hepatocellular and esophageal cancer [28]. Tumor infiltrating lymphocytes, especially CD8 cytotoxic T lymphocytes, could attenuate the metastatic potential of tumor cells by modifying tumor stroma and tumor cells. A standardized, powerful immune stratification system, the Immunoscore, was established to predict clinical outcomes. However, to the best of our knowledge, we are the first to apply tumor infiltrating lymphocytes in guiding the decision of adjuvant chemotherapy in stage I NSCLC patients. Our study shows that CD8+TILs are more valuable than traditional high risk features. Moreover, in the study of Arman et al., a higher degree of TILs within stage I non-small-cell lung cancer correlates with decreased risk of disease recurrence and improved DFS. They also suggested that TILs has particular appeal with regards to patient stratification as a means of guiding adjuvant chemotherapy. [29] Currently, adjuvant chemotherapy is the only therapy that is recommended by NCCN for stage I NSCLC patients with high risk of progression. What's more, previous studies demonstrated that TILs, PD-L1 and Foxp3+TILs were significant indicators for cisplatin based chemotherapy response. [30–32] It indicated that these parameters may aid in selecting patient populations that are most likely to benefit from cisplatin based chemotherapy. Based on this, patients with surgically resected stage I NSCLC showing low CD8+TILs could be considered for adjuvant chemotherapy, even if they have no high risk features.

Cancer immunotherapy is currently a very promising therapeutic strategy in NSCLC. Among those different immunotherapies, the monoclonal antibody blocking immune-checkpoint is currently considered the most popular approach. Such as Nivolumab, an anti PD-L1 antibody, has been approved for application in NSCLC by the U.S. Food and Drug Administration. Expression of PD-L1 is an important and widely-explored predictive biomarker to select patient subpopulations that will benefit from checkpoint blockades for PD-1/PD-L1 [33]. In our results, we analyzed PD-L1 expressions with IHC in surgically resected stage I NSCLC. To avoid the effects from cytotoxic agents, all patients received no adjuvant chemotherapy. Positive PD-L1 expressions were found in 19.8% patients in stage I NSCLC. PD-L1 expression is higher in men, squamous and poorly differentiated tumor. Our results were consistent with previous publications. PD-L1 positive expressions tend to be higher in stage I-II NSCLC than stage III-IV NSCLC, which is 20-36% in early stage NSCLC compared to 14-20% in advanced stage NSCLC [16, 34]. Additionally, PD-L1 positive expression is significantly higher in poorly differentiated tumor and squamous-cell NSCLC [35, 36]. In view of the positive correlation between PD-L1 expression and anti PD-1/PD-L1 treatment outcome, PD-L1 expression may guide immunotherapy strategies in defined subgroups. In our study, we didn't find any significant relationships between PD-L1 positive expressions and DFS or OS outcomes, which are inconsistent with the results of Cooper and Yang et al [17, 19]. Currently, the prognostic effect of PD-L1 in NSCLC patients remains controversial, ranging from negative to positive [18, 20, 37–39]. Some possible reasons for this are that 1) PD-L1 expression based on IHC were assessed by different anti-PD-L1 antibodies and staining methods (manual versus automated); 2) Different cut-off values to define PD-L1 positivity were applied, 1%, 5% and 10% were most used; and 3) PD-L1 expression is a dynamic process during cancer immune editing process [40]. To clarify the prognostic effects of PD-L1 in NSCLC, consistent detecting technology, cut-off value, time point and particularly defined stage and histology of NSCLC are needed.

Recently, a great interest in Foxp3+T cells led to studies of different type of cancers including NSCLC. Foxp3+T cells were thought to have the ability to suppress antitumor immune response by inhibiting the activity of cytotoxic T cells [41]. Based on our results, there were no significant correlations between Foxp3+TILs and patient outcomes. However, the combination of Foxp3+TILs and CD8+TILs was significantly associated with DFS. This is consistent with the studies of Chaputetal [42]. They found that TILs with CD8+^HIGH^/Foxp3+^LOW^ were significantly associated with improved 5y-OS in early stage NSCLC patients. This may be because Foxp3+T cell plays its immunosuppressive role effectively mainly through limiting the functions of CD8+T cell [41]. Jackute et al [43]. found that patients with high tumor infiltrating Foxp3 + CD4+ T cells tend to have prolonged overall survivals. There were also some studies indicating that the ratio between CD8+TILs and Foxp3+CD4+ TILs shows better prognostic values than either parameter alone [44]. In addition, it has been reported that a high frequency of Foxp3+ cells in regional lymph nodes is significantly associated with poor prognosis in patients with surgically resected stage I NSCLC [45]. In view of the above, we suggested that Foxp3+TILs may not be independently significant indications in NSCLC, but they may show some prognostic effects when combined with CD8+/CD4+TILs. Further study is needed to clarify the functions of Foxp3+TILs in NSCLC.

It is widely accepted that the tumor local immune-environment plays an important role in tumor proliferation, invasion and metastasis. To deliver personalized treatments, it is crucial to find an effective marker to monitor the tumor growth and therapy resistance. Unfortunately, we didn't find any correlations between CD8+TILs and peripheral circulating lymphocytes or any prognostic value of circulating lymphocytes. Since the systemic inflammatory response plays an important role in tumor progression, some inflammatory indicators, such as lymphocyte-to-monocyte ratio, neutrophil-to-lymphocyte ratio and platelet-to-lymphocyte, were found associated with patient prognosis in previous studies [46, 47]. Moreover, some immune-related cytokines were demonstrated effective predictors for a variety of tumors. Liao et al found a clear association between the Th17-related cytokine profile and the risk of NSCLC [48]. Forero et al. demonstrated that expression of the major histocompatibility complex (MHC)-II in triple negative breast cancer peripheral circulating tumor cells may be related to lower rate of relapse and enhanced progression free survival by triggering an antitumor immune response [49]. With the development of immunotherapy in cancers, more predictive markers are needed to monitor treatment effects and patient outcomes. CD8+TILs are effective prognostic factors for NSCLC patients. However, the monitoring power of TILs may be limited because the requisite biopsies are invasive and hard to repeat frequently. In the future, patients should receive more specific examinations based on gene, ribonucleic acid (RNA) or protein levels, not only the routine blood examination.

This study had limitations common to any retrospective research. In addition, we did not separately analyze TILs in different areas in the tumor microenvironment such as invasive margins and cancer cell nests. However, previous studies demonstrated that the density of infiltration of inflammatory cells is a more effective predictor than the type and location [50]. The tumor microenvironment involves complicated interactions between several types of infiltrating immune cells such as T and B cells, monocytes, dendritic cells, neutrophils, eosinophils, basophils, mast cells, and natural killer (NK) cells and the heterogeneous tumor cells themselves as well as the overexpressed checkpoints, which contribute to the regulation of antitumor immune responses under physiologic conditions [51]. The challenging for us is to find potential predictive markers to guide the treatments. In previous studies, CD3, CD4, CD45RO, CD8, Foxp3+T cells and Myeloid-derived suppressor cells (MDSCs) are the most common infiltrating immune cells tested in cancers. According to the results of a meta-analysis, CD8+TILs were more effective indicator of NSCLC prognosis than CD3+, CD4+TILs [52]. As the cytotoxic T cell, CD8+TILs are crucial in the effective phase of immune response. Moreover, Foxp3+TILs, as a group of suppressive T cells, have been extensively studied in cancers and some clinical studies indicating that increased Foxp3+TILs levels are negative prognostic factors [53]. There is increasing evidences showing that MDSCs are potent suppressors of anti-tumor T cell responses. However, MDSCs were not found increased in NSCLC and no correlations with prognosis. The previous data suggested that MDSCs may not be a suitable biomarker in NSCLC [53]. But beyond that, there are some other potential predictive infiltrating immune cells such as dendritic cells (DCs), which play an important role in antigen presentation and inducing T cells response. However, as a retrospective study and limited pathologic tissue of each patient, we failed to analyze the effects DCs-TILs in NSCLC. As a potential target of immunotherapies, DCs-TILs should be included in the future studies. Besides lymphocytes, a number of co-stimulatory molecules together to provide the second signals, of which PD-L1 and CTLA-4 are the most popular ones. Several anti PD-L1 antibodies have been approved for application in NSCLC. [33] As a potential predictive marker of immunotherapy, we analyzed PD-L1 expressions in NSCLC. However, little literatures reported expressions of CTLA-4 in cancers including lung cancers, head and neck cancers, melanoma and liver cancers. It suggested that CTLA-4 may not an effective prognostic indicator. IHC was used in our study because samples that have been exposed to fixatives (such as formalin or alcohol) are unacceptable for flow cytometry. In addition, IHC was most widely used in previous studies to detect the expressions of TILs and PD-L1. We choose IHC to make our results more consistent and comparable; fluorochrome-based signals last only 3 to 10 days; IF fluorochromes are light sensitive, so special care must be taken throughout the staining procedure to minimize loss of signal. The enzymes and most substrates used in IHC, on the other hand, are relatively light insensitive, and therefore the staining procedure can easily be performed on the open benchtop without any unusual precautions; since we have published several papers about IHC [54, 55], our group is very good at IHC and IHC analysis.

Last but not least, in our manuscript, positive control specimens for PD-L1 and Foxp3 IHC were created by human placenta and human colon cancer (Supplementary Figure S1). An isotype control was used as a negative control for each case stained for all markers to control for potential false positive staining. Based on these, we believe our results are reasonable and convincible.

The most direct and convincible way to demonstrate CD8+TILs is an effective indicator for adjuvant chemotherapy is to analyze the difference in DFS of low-CD8+TILs patients with and without adjuvant chemotherapy. We have to admit that this study had limitations common to any retrospective research. We could not demonstrate the conclusion directly as a clinical trial. However, according to our results, patients with high-CD8+TILs showed better DFS than patients with low-CD8+TILs, whatever the risk status. This indicated that patients with low-CD8+TILs should be considered for treatment intensification even if they don't have traditional high risk features. Currently, adjuvant chemotherapy is the only therapy that is recommended by NCCN for stage I NSCLC patients with high risk of progression. Therefore, we drew the conclusion that expressions of CD8+TILs in stage I NSCLC may guide adjuvant chemotherapy decisions.

In conclusion, CD8+TILs were significantly associated with DFS and OS in patients with surgically resected stage I NSCLC. CD8+TIL is a better predictor for DFS than traditional high risk features. Based on this, patients with surgically resected stage I NSCLC showing low CD8+TILs should be considered for adjuvant chemotherapy, even if they have no high risk features. Moreover, there's no correlation between PD-L1 expressions and patients' outcomes. PD-L1 tended to be highly expressed in men, squamous and well differentiated carcinoma. In addition, as a group of immunosuppressive lymphocytes, Foxp3+TILs alone didn't show any prognostic effects in patients with stage I NSCLC, but was associated with DFS when combined with CD8+TILs.

## MATERIALS AND METHODS

### Patients

Between January 2004 and January 2012, we retrospectively screened 126 patients who were pathologically diagnosed as stage I NSCLC in Shandong Cancer hospital. All of them received anatomical segmentectomy or standard lobectomy via open thoracotomy. Patients who underwent adjuvant or neoadjuvant chemotherapy or radiotherapy are excluded from this study. All patients signed informed consents. This study is approved by the scientific review and ethics committee of Shandong Cancer Hospital and Institute.

### Immunohistochemistry

Tumor specimens were obtained by surgery from 126 NSCLC patients. Surgically resected specimens were fixed in formalin and embedded in paraffin. Expressions of PD-L1 and Foxp3 were evaluated using immunohistochemistry (IHC). Four-um thick sections were deparaffinized, heated to 190^°^C for 5 minutes for antigen retrieval in ethylenediaminetetraacetic acid (EDTA) buffer pH 9.0. After cooling, endogenous peroxidase quenching was blocked by 3% hydrogen peroxidase for 5 minutes in room temperature (RT). Then the slides were blocked with 5% FBS(RT) for15minutes and incubated for 1 hour with primary antibodies against PD-L1 (M4424, Spring Bioscience, CA, 0.19ug/ml) or Foxp3 (M3974, Spring Bioscience, California, 0.48ug/ml). EnVision TM+ anti-rabbit HRP labeled polymer (Dako) was used as the secondary antibody staining for 30 minutes. Then 3, 3′-diaminobenzidine tetrahydrochloride was applied for color development in RT for 5 minutes, and sections were subsequently counterstained with haematoxylin. CD8 (ZM-0417, Beijing Zhongshan Golden Bridge Biotechnology Company, China) staining was performed according to standard automated protocols, which was performed by Leica Bond automated immunostainer (Leica) as described previously [25]. Positive control specimens for PD-L1 and Foxp3 IHC were created by human placenta and human colon cancer (Supplementary Figure S1). An isotype control was used as a negative control for each case stained for all markers to control for potential false positive staining.

All IHC results were evaluated by 2 independent pathologists who were blinded to patients' clinical outcomes. Foxp3 expressions were evaluated according to the average number of positively-stained cells in 5 randomly and averagely selected high-power fields (HPF) in each case [23]. The expressions of CD8+TILs were evaluated according to the percent of CD8 positive staining lymphocytes. To subdivide patients, we calculated the median counts of Foxp3+TILs andCD8+TILs as the cut-off points. PD-L1 expression was defined as positive if greater than or equal to 5% of tumor cells had membranous staining, as used previously [56].

### Statistical analysis

The correlations between immune markers expressions and clinicopathologic characteristics were evaluated using Chi square tests. Overall survival (OS) was defined as the time between operation and death due to any cause. Disease free survival (DFS) was defined as the date of operation to the date of patients showing any signs or symptoms of local or distant recurrence. Patient survival curves were performed by using the Kaplan–Meier method and compared by log-rank test. Univariate Cox proportional hazard models were used to identify factors significantly associated with DFS and OS. Multivariate Cox regression analysis was constructed with variables that were significant in univariate analysis. The correlations between CD8+TILs and peripheral blood lymphocytes were evaluated with Pearson correlation analysis. All analyses were two-sided and p values < 0.05 were considered statistically significant. Statistical analyses were performed using Statistical Product and Service Solutions (IBMSPSS17.0).

## SUPPLEMENTARY FIGURES


