# Supplementary material for: Expressions of CD8+TILs, PD-L1 and Foxp3+TILs in stage I NSCLC guiding adjuvant chemotherapy decisions
Source: Oncotarget. 2016 Sep 1;7(39):64318–29. doi: 10.18632/oncotarget.11793 (PMC5325445; doi:10.18632/oncotarget.11793)
Supplement: Supplementary file 1 [file oncotarget-07-64318-s001.pdf]

Expressions of CD8+TILs, PD-L1 and Foxp3+TILs in stage I NSCLC guiding adjuvant chemotherapy decisions

SUPPLEMENTARY FIGURES

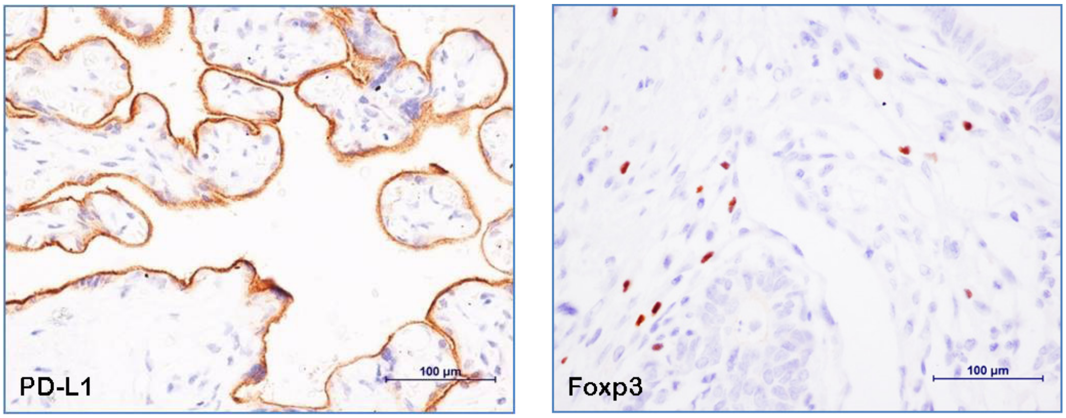

Supplementary Figure S1: Positive control specimens for PD-L1 and Foxp3 IHC in human placenta and human colon cancer, respectively.

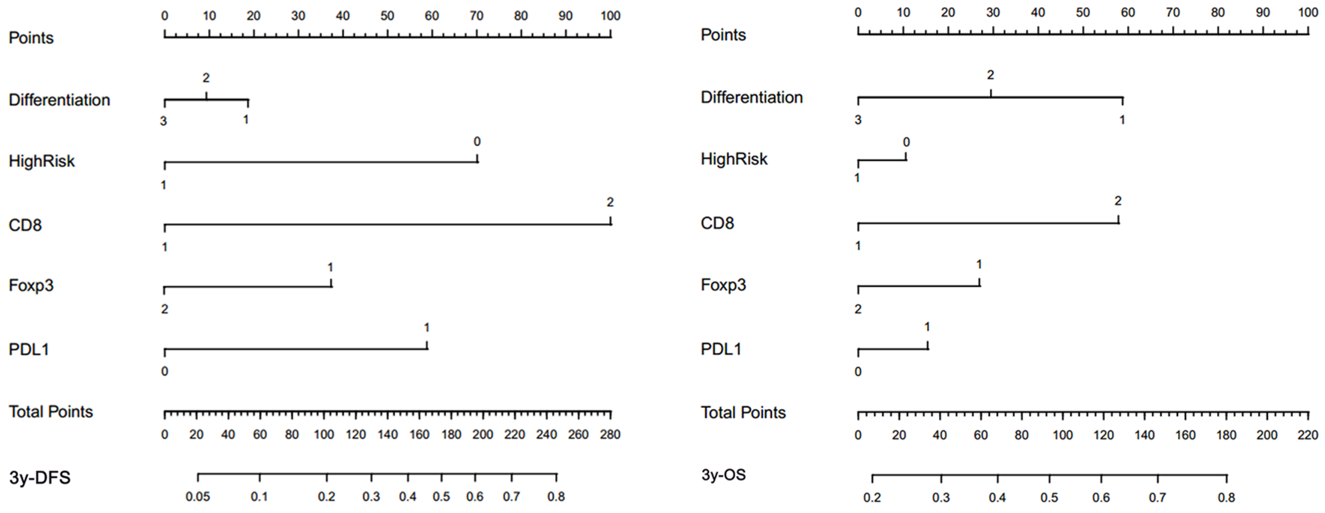

Supplementary Figure S2: NSCLC DFS and OS nomograms. (To use the nomograms, an individual patient's value is located on each variable axis, and a line is drawn upward to determine the number of points received for each variable value. The sum of these numbers is located on the Total Points axis, and a line is drawn downward to the survival axes to determine the likelihood of 3y-DFS or 3y-OS).
